# Evaluation of a modified version of the Posttraumatic Growth Inventory-Short Form

**DOI:** 10.1186/s12874-017-0344-2

**Published:** 2017-04-20

**Authors:** Navjot Kaur, Ben Porter, Cynthia A. LeardMann, Laura E. Tobin, Hector Lemus, David D. Luxton, for the Millennium Cohort Study Team, Richard Armenta, Lauren Bauer, Deb Bookwalter, Anna Bukowinski, Adam Cooper, James Davies, Alex Esquivel, Dennis Faix, Susan Farrish, Toni Rose Geronimo, Gia Gumbs, Isabel Jacobson, Claire Kolaja, Joyce Kong, William Lee, Kyna Long, Denise Lovec-Jenkins, Gordon Lynch, Rayna Matsuno, Danielle Mitchell, Kristin Motylinski, Anna Nagel, Chiping Nieh, Chris O’Malley, Serguey Parkhomovsky, Anet Petrosyan, Chris Phillips, Teresa Powell, Rudy Rull, Beverly Sheppard, Steven Speigle, Daniel Trone, Jennifer Walstrom, Paul Amoroso, Edward Boyko, Gary Gackstetter, Greg Gray, Tomoko Hooper, Margaret Ryan, Tyler Smith, Timothy Wells

**Affiliations:** 1 Deployment Health Research Department, Naval Health Research Center, 140 Sylvester Rd., San Diego, CA 92106-3521 USA; 2 Henry M. Jackson Foundation for the Advancement of Military Medicine, Inc., 6720A Rockledge Dr. #100 Bethesda, Baltimore, MD 20817 USA

**Keywords:** Psychological well-being, Posttraumatic Growth Inventory, Military, Psychometrics

## Abstract

**Background:**

Posttraumatic growth is the positive change resulting from traumatic experiences and is typically assessed with retrospective measures like the Posttraumatic Growth Inventory (PTGI). The PTGI was designed to include reference to a specific traumatic event, making it difficult to implement, without change, in prospective survey studies. Thus, a modified Posttraumatic Growth Inventory–Short Form (PTGI-SF) was included in a large prospective study of current and former U.S. military personnel. The current study provides preliminary psychometric data for this modified measure and its ability to assess psychological well-being at a single time point.

**Methods:**

The study population (*N* = 135,843) was randomly and equally split into exploratory and confirmatory samples that were proportionately balanced on trauma criterion. Exploratory factor analysis and confirmatory factor analysis (CFA) were performed to assess the psychometric validity of the modified measure. The final model was also assessed in a subset of the confirmatory sample with a history of trauma using CFA.

**Results:**

Results supported a single-factor model with two additional correlations between items assessing spirituality and items assessing compassion/appreciation for others. This model also fits among the subset with a history of trauma. The resulting measure was strongly associated with social support and personal mastery.

**Conclusions:**

The modified PTGI-SF in this study captures psychological well-being in cross-sectional assessments, in addition to being able to measure posttraumatic growth with multiple assessments. Results indicate that the modified measure is represented by a single factor, but that items assessing spirituality and compassion/appreciation for others may be used alone to better capture these constructs.

## Background

Posttraumatic growth refers to the positive psychological change (e.g., stronger spiritual faith or more compassion) after the experience of traumatic events [1–3]. Posttraumatic growth has been documented among persons who have faced varied types of trauma, including the loss of a loved one [4], cancer diagnosis [5], and the experience of war [6–8]. The Posttraumatic Growth Inventory (PTGI) is a 21-item measure of positive growth that uses a 6-point Likert-type response scale [2]. The 21-item scale has been shown to fit a five-factor structure consisting of (a) relating to others, (b) new possibilities, (c) personal strength, (d) spiritual change, and (e) appreciation of life [2, 9]. The full scale has been abbreviated into the 10-item Posttraumatic Growth Inventory–Short Form (PTGI-SF) in order to make administration of the measure more feasible in contexts where time or space is limited [10]. Ten items from the PTGI (two from each subscale) were selected for inclusion in the PTGI-SF [10]. Similar to the full measure, the PTGI-SF has been used to examine posttraumatic growth after different types of traumatic experiences, including negative medical diagnoses [11], life stressor events [12], acts of terrorism [13], and exposure to war [14].

The full scale and short form versions ask individuals to report on growth by using retrospective self-reported items. However, this method of measurement has been criticized [15]. The primary criticism is that it may be difficult for individuals to accurately recall and compare their pre-trauma status with their current status. Furthermore, perceptions of growth following traumatic events may be distorted by known psychological processes (e.g., creating positive illusion) making it difficult to measure actual growth [15, 16]. In order to examine the validity of the full scale, Frazier and colleagues adapted phrasing of the items to refer to feelings over the last 2 weeks, rather than since a traumatic event [15]. Respondents completed these items at two time points, which were used to prospectively assess change from pre- to post-trauma. The study found that objective changes measured with this adapted scale were unrelated to retrospective perceptions of change assessed using the PTGI at the second time point, indicating that the PTGI may not accurately capture growth following a traumatic event [15]. Due to these concerns about the PTGI, researchers have been encouraged to conduct longitudinal studies to prospectively assess posttraumatic growth [17, 18].

Launched in 2001, the Millennium Cohort Study is the largest prospective epidemiological study in U.S. military history. It was designed to evaluate the impact of military service on the health of U.S. service members. Health status, occupational factors, and life experiences are assessed approximately every 3 years using a self-report questionnaire [19, 20]. A modified version of the PTGI-SF, similar to that created by Frazier et al. [15], was added to the Millennium Cohort Study survey during the 2011–2013 survey cycle. The wording was altered to assess an individual’s current state to remove the retrospective recall component and to collect information on all participants, regardless of trauma. Similar to other prospective posttraumatic growth studies [15, 18], the intent of these modifications was to be able to prospectively measure growth using longitudinal data from subsequent surveys. However, given the content of the items, the modified version of the PTGI-SF is likely to also capture psychological well-being when assessed using a cross-sectional design.

Although Frazier and colleagues have examined a similar measure [15], to the authors’ knowledge, no study has examined the psychometric properties of a PTGI scale modified this way. The current study examined the factor structure of this modified PTGI-SF instrument. In addition, convergent and divergent validity of the resulting measure was established with other measures contained within the Millennium Cohort Study survey.

## Methods

### Participants

Since 2001, the Millennium Cohort has enrolled over 200,000 participants during four sequential enrollment cycles. The Millennium Cohort procedures were approved by the Institutional Review Board at the Naval Health Research Center. All participants provided voluntary, informed consent. The study population for the current analyses was limited to participants who completed a questionnaire during the 2011–2013 survey cycle (*n* = 138,949). Additionally, participants missing responses on physical assault, sexual assault, and other items designed to assess traumatic exposures were excluded (final *N* = 135,843; 97.7% of all survey cycle participants). A stratified random sampling technique was used to separate participants into exploratory and validation samples. Because trauma may influence the underlying structure of the measure, strata of participants with every possible combination of history of physical trauma, sexual trauma, and other traumatic experiences were created and randomly divided into the two equal samples. The exploratory sample contained 67,921 participants, and the validation sample contained 67,922 participants.

### Measures

As coined by Frazier and colleagues, the “current standing” PGTI-SF (C-PTGI-SF), which measures the current state of each item regardless of past traumatic experience [15], was first introduced as a part of the Millennium Cohort survey during the 2011–2013 cycle. In addition to the modifications mentioned, an additional item from the full PTGI (“I have compassion for others”) was included in the 11-item C-PTGI-SF to provide more information about positive social growth. Participants responded to the prompt, “Indicate the degree to which the following statements are true in your life…” on a 7-point Likert-type scale from 0 (*Not at all)* to 6 (*To a very great degree*) for items such as “I prioritize what is important in life” and “I have religious faith.” The items showed strong internal consistency (*α* = 0.92). See Table 2 for a complete list of the items.

To ensure equal representation of traumatic experiences in the exploratory factor analysis (EFA) and confirmatory factor analysis (CFA) sample, data from all survey cycles were used to assess the history of traumatic experiences. If a participant endorsed a trauma on any survey, he or she was categorized as exposed to that trauma. The sexual assault and physical assault items were individual statements with “yes” or “no” response options. “Other traumatic experiences” were ascertained using five items with a three-level response option for zero, one, or more than one exposure. These five items refer to traumatic events that are likely to occur in combat (e.g., witnessing a person’s death due to war, disaster, or a tragic event; seeing dead bodies); however, some individuals may have experienced such events outside of combat (e.g., paramedics), so responses are not limited to only wartime experiences. Participants were considered to have had “other traumatic experiences” if one or more of the five items were endorsed, regardless of the circumstance of the traumatic experience.

Demographic (e.g., gender, birth year, education, marital status, race/ethnicity) and military information (e.g., military pay grade, service component, branch of service) were assessed at the 2011–2013 survey cycle using a combination of self-report and administrative records. Deployment status was based on data obtained from the Defense Manpower Data Center and self-reported combat experiences in support of the operations in Iraq and Afghanistan since 2001. Those deployed were considered to have experienced combat if they reported personally witnessing death, prisoners of war, refugees, maimed soldiers, or physical abuse.

Hypertension, social support, and personal mastery were examined to establish convergent and divergent validity. The hypertension item response options were “yes” or “no” to indicate whether an individual had received a diagnosis by a medical professional. The social support item asked, “In the last 4 weeks, how well have your family and friends supported you?” and was scored with a 5-point Likert scale response (0 = *Not at all*, 4 = *Extremely*). Three items from the Pearlin Mastery Scale (e.g., “What happens to me in the future mostly depends on me”) were also used with response options ranging from 0 (*Strongly disagree)* to 5 (*Strongly agree*) [21]. Personal mastery was measured by the mean score of these three items.

### Statistical analysis

Demographic characteristics were summarized for the participants who completed the survey during the 2011–2013 survey cycle. EFA using iterated principal factor analysis was conducted on the exploratory sample. An oblique rotation method was used, allowing factors to be correlated [22, 23]. The Kaiser criterion, scree plot, and interpretability of results were used to determine the number of factors to retain [23]. CFA was conducted using the validation sample and separately with a subset of the validation sample that had experienced trauma. Model fit was assessed with the comparative fit index (CFI), Tucker-Lewis index (TLI), root mean square error of approximation (RMSEA), and standardized root mean square residual (SRMR). Cutoff values for these indices vary, however, generally high CFI and TLI values along with low RMSEA and SRMR values indicate a good model fit [24]. Differences between the models were assessed using the Akaike information criterion (AIC) because AIC is sensitive to differences in changes in degrees of freedom [24].

Convergent validity was examined through correlations with the social support item and three items from the Pearlin Mastery Scale. Divergent validity was assessed using hypertension. Hypertension was selected because it was expected to affect physical health but not psychological well-being.

CFA models were evaluated using Mplus Version 7.3 (Muthén & Muthén, Los Angeles, CA). All other analyses were performed using SAS 9.3 (SAS Institute Inc., Cary, NC).

## Results

A majority of the participants were non-Hispanic White (72.9%), male (70.2%) and born after 1979 (50.4%). The prevalence of individuals with posttraumatic stress disorder in the study population was 10.7%. About 8% of the population reported a history of sexual assault; similarly about 9% of the population reported a history of physical assault. Nearly half of the population had experienced other traumatic experiences (44.6%). Demographic information for study participants is summarized in Table 1.

**Table 1:** Demographic information for millennium cohort study participants, 2011–2013 survey cycle (*N* = 135843)

Characteristics	Population *n* (%)
Gender	
Men	95,321 (70.2)
Women	40,522 (29.8)
Birth year	
Pre-1960	13,644 (10.0)
1960–1969	23,147 (17.0)
1970–1979	30,528 (22.5)
1980+	68,524 (50.4)
Education	
High school diploma or less	15,640 (11.5)
Some college^a^	67,803 (49.9)
Bachelor’s degree	30,838 (22.7)
Master’s degree/PhD	21,560 (15.9)
Marital status	
Never married	28,590 (21.1)
Married	87,472 (64.4)
Divorced^b^	19,781 (14.6)
Race/ethnicity	
Non-Hispanic White	98,919 (72.9)
Non-Hispanic Black	14,259 (10.5)
Other	22,600 (16.6)
Military pay grade	
Commissioned or Warrant officer	31,251 (23.0)
Enlisted	104,591 (77.0)
Service component	
Active duty	79,092 (58.2)
Reserve	56,751 (41.8)
Branch of service	
Army	60,638 (44.6)
Navy/Coast Guard	24,160 (17.8)
Marines	10,988 (8.1)
Air Force	40,057 (29.5)
Deployment status	
Nondeployed	51,162 (37.7)
Deployed without combat	42,562 (31.3)
Deployed with combat	42,119 (31.0)
Posttraumatic stress disorder^c^	
Yes	14,362 (10.7)
No	120,126 (89.3)
Ever suffered a violent assault	
Yes	12,101 (8.9)
No	123,742 (91.1)
Ever suffered a sexual assault	
Yes	10,617 (7.8)
No	125,226 (92.2)
Other traumatic experiences^d^	
Yes	60,525 (44.6)
No	75,318 (55.4)

Due to rounding, all percentages may not add up to 100. Not all characteristics had a population of 135,843 due to missing values

^*a*^Some college includes completing an associate degree

^*b*^Divorced includes those who have annulled their marriage, legally separated, and have been widowed

^*c*^Posttraumatic stress disorder (PTSD) was ascertained by the 17-item PTSD Checklist–Civilian Version.^28^ Participants were considered to screen positive for PTSD if they met the criteria that correspond to DSM-IV diagnostic criteria.^29^

^*d*^Other traumatic experiences was defined as participants who experienced traumatic events captured by the measure (e.g., witnessing a person’s death due to war, disaster, or a tragic event; seeing dead bodies)

Basic descriptive statistics for each item are provided in Table 2. All items were likely to be endorsed by each participant as shown by the means and medians of all the items being above the midpoint of each scale. However, each item had a standard deviation greater than or equal to 1.0 indicating sufficient variability in each item. Each item was strongly correlated with the total scale (*r*’s > .52).

**Table 2:** Item-level descriptive statistics across total sample (*n* = 135,843)

Item	Mean (SD)	Median [IQR]	Item-total correlation
I am able to do good things with my life.	4.0 (1.0)	4 [4–5]	.76
I have an appreciation for the value of my own life.	4.2 (1.0)	4 [4–5]	.69
I know that I can handle difficulties.	4.0 (1.0)	4 [4–5]	.64
I established a path for my life.	3.5 (1.3)	4 [3–4]	.73
I prioritize what is important in life.	3.8 (1.0)	4 [3–5]	.59
I have a sense of closeness with others.	3.5 (1.3)	4 [3–4]	.76
I’m stronger than I thought I was.	3.6 (1.3)	4 [3–5]	.66
I have learned a great deal about how wonderful people are.	3.1 (1.5)	3 [2–4]	.70
I have compassion for others.	3.7 (1.2)	4 [3–5]	.64
I have religious faith.	3.1 (1.7)	4 [2–5]	.52
I have understanding of spiritual matters.	3.5 (1.4)	4 [3–5]	.66

### Exploratory factor analysis

**Fig. 1:**
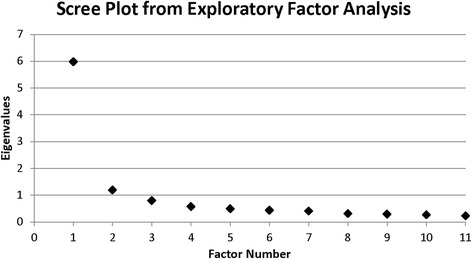
Scree plot of eigenvalues for exploratory factor analysis

The Kaiser criterion indicated two factors with eigenvalues of 6.0 and 1.2. The scree plot, a method to depict the eigenvalues and assess factor separation (Fig. 1), suggested a possible range of one to three factors. A two-factor EFA was examined first since it was supported both by the Kaiser criterion and scree plot (Table 3). The two-factor EFA identified one factor with six strongly loading items (loadings > .60) while the other factor had only two items strongly loading onto it (“I have religious faith” and “I have an understanding of spiritual matters”). Two of the remaining three items (“I have compassion for others” and “I have learned a great deal about how wonderful people are”) cross-loaded onto both factors. The correlation between the two factors was 0.54.

**Table 3:** Factor loadings of exploratory factor analysis with two factors (*n* = 65,306)

Item	Factor loadings	
	Factor 1	Factor 2
I am able to do good things with my life.	0.90	
I have an appreciation for the value of my own life.	0.82	
I know that I can handle difficulties.	0.80	
I established a path for my life.	0.77	
I prioritize what is important in life.	0.69	
I have a sense of closeness with others.	0.68	
I’m stronger than I thought I was.	0.57	
I have learned a great deal about how wonderful people are.	0.51	0.32
I have compassion for others.	0.46	0.29
I have religious faith.		0.87
I have understanding of spiritual matters.		0.69

Factors with an absolute loading <0.20 not shown

One known limitation of two-item factors is that they are locally underidentified in structural equation models [24, 25]. Additionally, cross-loadings can reduce the interpretability of results and can be the result of an underlying factor [24]. To avoid these problems, additional solutions were investigated. However, the three-factor solution posed similar problems (Table 4). The EFA for the three-factor model identified one factor with five strongly loading items, while the other two factors had only two items strongly loading on each. The items “I have religious faith” and “I have an understanding of spiritual matters” loaded on the second factor, while “I have compassion for others” and “I have learned a great deal about how wonderful people are” loaded on the third factor. The remaining two items—“I have a sense of closeness with others” and “I’m stronger than I thought I was”—cross-loaded onto two different factors. The three factors were highly correlated with each other (*r* = 0.67, 0.57, and 0.52, respectively).

**Table 4:** Factor loadings of exploratory factor analysis with three factors (*n* = 65306)

Item	Factor loadings		
	Factor 1	Factor 2	Factor 3
I am able to do good things with my life.	0.88		
I have an appreciation for the value of my own life.	0.80		
I know that I can handle difficulties.	0.77		
I established a path for my life.	0.67		
I prioritize what is important in life.	0.70		
I have a sense of closeness with others.	0.43	0.40	
I’m stronger than I thought I was.	0.39	0.29	
I have learned a great deal about how wonderful people are.		0.97	
I have compassion for others.		0.58	
I have religious faith.			0.88
I have understanding of spiritual matters.			0.79

Factors with an absolute loading <0.20 not shown

Given the high alpha, large initial eigenvalue, and high correlations between factors, a single-factor model was also examined. All but one item loaded onto this factor with a factor loading greater than 0.60 (Table 5). The item “I have religious faith” had a moderate factor loading of 0.51. The study team chose to proceed with the single-factor model because it did not have cross-loading items or factors with only two items and had a strong association with all of the items. The study team named this factor positive psychological well-being.

**Table 5:** Factor loadings of exploratory factor analysis with one factor (*n* = 65306)

Item	Factor loadings
	Factor 1
I am able to do good things with my life.	0.82
I have an appreciation for the value of my own life.	0.76
I know that I can handle difficulties.	0.70
I established a path for my life.	0.78
I prioritize what is important in life.	0.63
I have a sense of closeness with others.	0.80
I’m stronger than I thought I was.	0.70
I have learned a great deal about how wonderful people are.	0.73
I have compassion for others.	0.66
I have religious faith.	0.51
I have understanding of spiritual matters.	0.64

### Confirmatory factor analysis

**Fig. 2:**
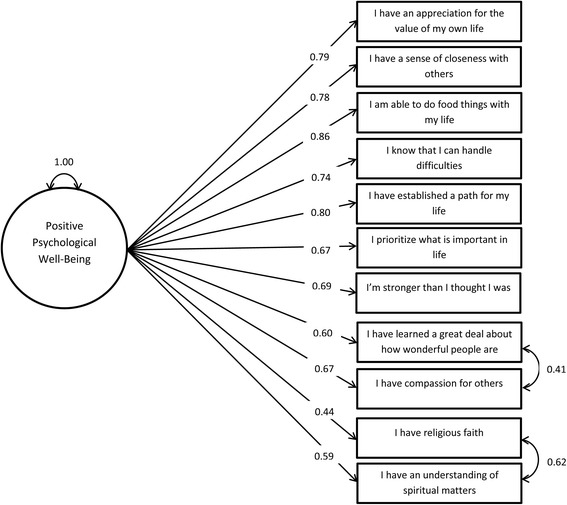
Results of the confirmatory factor analysis. Confirmatory factor analysis yielded the final model, a one-factor model with additional covariances between the “faith” and spirituality” items and between the “compassion” and “wonderful” items. Residual variances were estimated but not included in the graphical representation

A single-factor CFA was conducted using the validation sample. The single-factor model demonstrated poor fit (AIC = 1,975,061; CFI = 0.910; TLI = 0.884; SRMR = 0.048; RMSEA = 0.090). To improve fit, residual covariances were added to the model to account for the additional associations between the two pairs of items from the three-factor EFA: (a) “I have religious faith” and “I have an understanding of spiritual matters,” and (b) “I have compassion for others” and “I have learned a great deal about how wonderful people are.” This model had better fit relative to the single-factor model, as seen by a reduction in AIC and moderate fit overall (AIC = 1,964,099; CFI = 0.936; TLI = 0.916; SRMR = 0.044; and RMSEA = 0.076). Figure 2 depicts the final selected model. The residual covariances of the religiosity items and the feelings toward others items equated to correlations of 0.86 and 0.69, respectively. A CFA using a subset of the validation sample that had experienced trauma was also conducted with the model depicted in Fig. 2. The fit indices within this model are comparable to those of the larger validation sample (CFI = 0.936; TLI = 0.916; SRMR = 0.045; RMSEA = 0.076).

### Convergent and divergent validity

Pearson correlation coefficients were calculated between a total score calculated from the sum of all 11 C-PTGI-SF items and constructs identified a priori to establish validity. All correlations were significant at *p* < .0001. Hypertension (*r* = −0.06) was weakly correlated with C-PTGI-SF. Social support (*r* = 0.42) and personal mastery (*r* = 0.42) were significantly and positively correlated with the C-PTGI-SF.

## Discussion

A single-factor model emerged as the best fit for these 11 items on the Millennium Cohort survey. EFAs were conducted for models with factors ranging from one to three. Because the two- and three-factor models did not show clear factor separation, the study team examined and selected a single-factor model, which was also supported by the scree plot. This model was subsequently examined using CFA. Residual covariances were included to prevent convergence problems and estimation errors associated with estimating factors with only two indicators. Additionally, they maintain the idiosyncratic association between the two items assessing faith and spirituality and the two items assessing compassion and appreciation for others. It is important to note that these adjustments were made from the results of the exploratory sample and were not discovered using modification indices in the confirmatory sample, which can result in misspecified final models [26, 27]. The current study indicates the utility of the C-PTGI-SF to capture positive psychological well-being at a single time point. Further, the C-PTGI-SF assessed before and after a traumatic event can be used to assess conventional posttraumatic growth.

Previously, the PTGI-SF was developed by Cann and colleagues using the following process to pare down the 21-item PTGI [10]. The strongest loading items from the appreciation of life and personal strength factors and the two items in the spiritual change factor were included as part of the PTGI. The two items from the remaining two factors (relating to others and new possibilities) were selected to expand the scope of the inventory because the strongest loading items of these factors were deemed redundant with items already included from the three other factors [10]. Those items were preferentially selected. Although the current study only found a single factor for the C-PTGI-SF, it is possible that had all the items from the long form been assessed, the previously discovered five-factor model of the PTGI would have separated out more cleanly. While subanalyses examined a subgroup consisting of only those who had experienced trauma, the main analyses of the current study differ from previous studies due to modifications to the scale and the study population, which included participants who had not experienced trauma.

Some evidence for subfactors in the two- and three-factor EFAs was observed, with two items strongly loading onto the second and third factors. However, these factors contributed to cross-loading among other items and were highly correlated with the main factor, which explains why these items factored well in the single-factor EFA. However, the two strong residual covariances suggest that these item pairs (spirituality items and compassion/appreciation for others items) could be used alone in future research projects.

Divergent validity was established using hypertension, which was weakly associated with the C-PTGI-SF. Hypertension was selected because its diagnosis was not expected to be associated with substantially decreased well-being. Additionally, convergent validity was established using social support and personal mastery. These items were selected since they also measure facets of positive personal and social well-being that the C-PTGI-SF captures. Both were moderately correlated with the C-PTGI-SF, demonstrating that the C-PTGI-SF is similar to, yet also distinct, from these two concepts.

An artifact of the data is that the factor structure from the EFAs did not show distinct factor separation and thus required additional interpretation and decision making to select a factor structure. However, while the factor structure was not initially well-defined, information provided by the EFAs was used to improve the fit for the final model without the use of modification indices. Also, the final model only exhibited moderate fit, findings similar to previous CFAs of the PTGI-SF [12, 14].

While this study has limitations, it also has notable strengths. The study had a very robust sample size and was representative of the military population as a whole, making the results generalizable across the military [28]. The large sample size allowed for the creation of exploratory and confirmatory samples, which prevent misspecification arising from fitting a model to available data. Additionally, with the large sample size, the estimates found in the current study are highly stable.

## Conclusions

The current study suggests the C-PTGI-SF may be used in cross-sectional and longitudinal studies. In addition to assessing positive psychological well-being, the item pairs allow for religiosity and relating to others to be examined individually as well. The modifications of the PTGI-SF make it more suitable for use with a large cohort study because such a prospective design does not allow for the identification of an index trauma. Furthermore, previous studies have questioned whether the PTGI can actually measure posttraumatic growth because the measure is based on retrospective self-report [29]. The modifications of the C-PTGI-SF instrument removed the retrospective report requirement of the measure. This allows well-being to be examined cross-sectionally and it makes it possible to objectively assess posttraumatic growth using data from two or more time points.

## Abbreviations

AIC: Akaike information criterion
CFA: Confirmatory factor analysis
CFI: Comparative fit index
C-PTGI-SF: Current standing posttruamtic growth inventory- short form
EFA: Exploratory factor analysis
IQR: Inter-quartile range
PTGI: Posttraumatic growth inventory
PTGI-SF: Posttraumatic growth inventory- short form
RMSEA: Root mean squared error of approximation
SD: Standard deviation
SRMR: Standardized root mean square residual
TLI: Tucker-Lewis index
U.S.: United States
